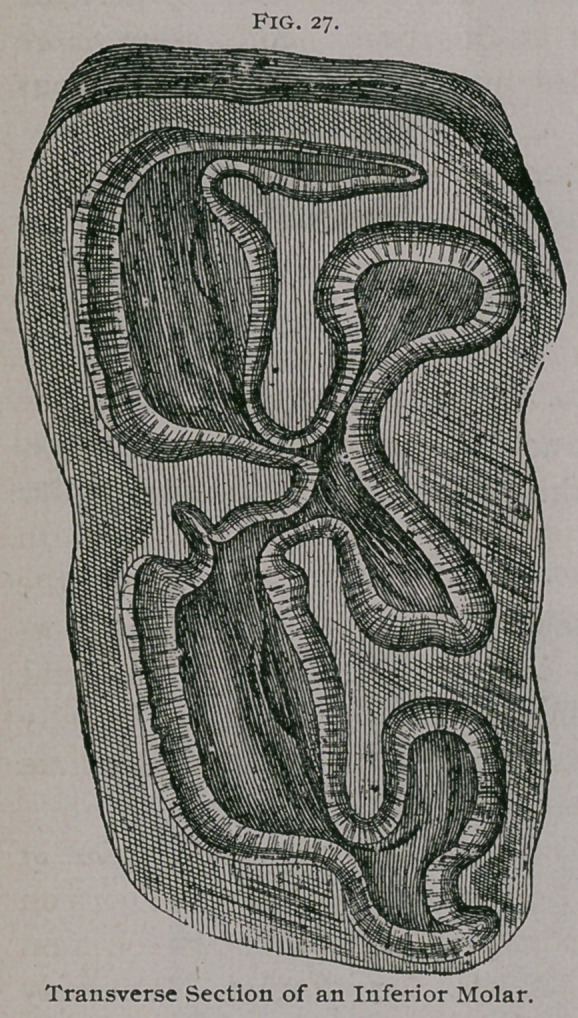# Age of the Horse, Ox, Dog, and Other Domestic Animals

**Published:** 1890-05

**Authors:** R. S. Huidekoper

**Affiliations:** Veterinarian


### AGE OF THE HORSE, OX, DOG, AND OTHER DOMESTICATED ANIMALS.

By R. S. Huidekoper, M.D., Veterinarian.

[*Continued from page 241.*]

### MOLARS.

I. Premolars—Wolf Teeth.—These teeth, which were first described by Daubenton, are not found in all horses. They occur much more frequently in the upper jaw than in the lower one, and are rarely found in both in the same animal. Girard says that they usually appear about the tenth month and drop out at the time of the eruption of the permanent molars. The alveola are usually directly in front of those of the first molars. They are, however, frequently a half inch or more in front of the others and often remain until the animal has reached an advanced age. Their presence renders the dental formula slightly variable (44 for the horse and 40 for the mare). The wolf teeth are elongated and slightly curved on their long axis ; they resemble a good deal the incisive tooth of a carnivora, from which, perhaps, comes their common name. Their roots are unicuspid. Girard describes supplementary post-molars, which, however, are not recorded by other authors, and were probably anomalous teeth.

II. Molars.—The molars fill in the sides of the dental arch ; like the incisors, they appear in two groups, the temporary and permanent teeth.

### *A. Molars of First Dentition.*

The *deciduous*, *temporary*, *milk*, or *molars of first dentition*, are twelve in number, six in each jaw, three on each side. By some curious error, the father of veterinary anatomy, Carlo Ruini, 1598, and his immediate followers, considered that there were but eight temporary molars.

These teeth are strong, short and have the general form of quadrangular prisms, except the first in each row, which is triangular.

FIG. 20.

The Three Superior Temporary Molars (right). F I., Internal Face ; F E., External Face.

[Goubaux and Barrier.]

The anterior and posterior faces are smooth ; in the upper jaw the first tooth has three longitudinal canals on its external face, while the second and third teeth have two ; the internal faces are irregular, slightly concave in their long axis and show canals very much less marked than those on the outside.

The inferior teeth on their external face have a single gutter, which is deepest in the first tooth, and most shallow in the last; on their internal face they are irregularly grooved. All of the milk molars have a constriction or neck separating their crowns from their roots. They have each two roots, one anterior and one posterior. These are strong, convex outside and concave inside;

FIG. 21.

The Three Inferior Temporary Molars (right side). F.I., Internal Face; F.E., External Face.

[Goubaux and Barrier.]

each is hollowed by an opening which reaches into the tooth itself. Just before being forced out by the permanent teeth the roots are sometimes divided into little eminences by the pressure of the irregularities on the crowns of the latter. The free extremities of the virgin milk molars are irregular and covered with ridges and cavities, but the external border of the superior teeth and the internal border of the inferior teeth are always longer than the opposite border, so that they present two oblique planes, which become more marked as the teeth wear down. As the animal becomes older, the molars wear down until they are reduced to little thin plates which fit close to, or cap, as it were, the crowns of the replacing molars, by which they are finally forced out from the jaw.

*B. Molars of Second Dentition—Permanent Molars.*

These are twenty-four in number, twelve in each jaw, six on each side. They are designated numerically from in front to behind, as the first, second, etc. The three first, which replace the temporary molars, are known as *pre-molars*, and the last three as the *post-molars.* Each row of six molars forms a branch of the dental arch, which in the upper jaw is slightly convex to the outer side, while in the lower jaw it is perfectly straight, with the anterior extremities inclined toward the other, so that they form a sort of V, and are overlapped by those of the upper jaw.

The permanent molars have the shape of quadrangular prisms, flattened from side to side, except the first and sixth, which are triangular.

*a. Superior Molars.*—The posterior faces are almost smooth, except in the sixth molar, where the face is replaced by a blunt edge; the anterior faces are also smooth, except that of the first molar, which is replaced by a sharp border.

The external faces have two longitudinal gutters separated by a ridge; the first molar has three gutters with two ridges; the internal faces have two shallow gutters on the first molar and one on each of the others, which is closer to the posterior border of the teeth in the last ones. There is no neck or distinctive line separating the crown or free extremity from the roots or imbedded extremity of the permanent molars. The free extremity is triangular in the first and sixth teeth and quadrangular in the others. In the virgin tooth it has an irregular surface resembling an Old English B with the loops looking toward the inside and surrounding cavities, which are more or less filled with cement; to the anterior loop is attached a small secondary loop. The external border is always longer than the internal. As the tooth wears, and the table is established, the cavities disappear. At first the embedded

FIG. 22.

Right-hand Inferior Molars of a Horse 6 to 7 years old.

FIG. 23.

Right-hand Superior Molars of a Horse 6 to 7 years old.

portion is hollowed by cavities, which reach the body of the tooth itself, and contain the papilla or pulp, formed principally of blood-vessels and nerves; later, these resolve into distinct roots, three each for the first and sixth molars and four each for the others. If the head is placed horizontally, the first molar is found embedded in a vertical position, while the others incline somewhat from below to above and from in front to behind.

The superior molars are implanted in alveolar cavities, prismatic like the teeth and separated from each other by bony septa, which are thin at their free borders and thicker above. The bottoms of the three last project into the maxillary sinuses, while those of the three first are in the superior maxillary bone itself.[1]

*b. Inferior Molars.*—The anterior and posterior faces are smooth, except the posterior of the sixth tooth and the anterior of the first tooth, which are replaced by sharp edges. The

FIG. 24.

A Right Superior Molar, taken from the Dental Follicle. [Goubaux and Barrier.]

A.—View from free end. B.—View from the embedded portion.

a, anterior infundibula; a′, posterior infundibula; e, external border of the infundibula; i, internal border of the infundibula; c, accessory column of the anterior infundibula.

external faces have one longitudinal gutter in the first five teeth and two in the sixth tooth. The internal faces have three gutters in the first and sixth teeth and a variable number in the others. The free extremity is triangular in the first and sixth molars and quadrilateral in the others; but these are narrower from side to side than from in front to behind. They are longer on their internal border than on the external, and have the Old English B turned with the loops outward. The embedded portion is bicuspid in the first five teeth and unicuspid in the sixth. The divisions of the roots diverge; each contains a cavity for the pulp.

[1] In very old horses the sinus may reach the root of the third or even second tooth.

### *C. Development and Structure of the Molars.*

In the superior molars the papilla of the enamel in the germinal sack is double and penetrates into the tooth, forming,

FIG. 25.

Grinding Table of a Superior Molar (left).

Table of an Inferior Molar (right).

practically, two infundibula, one anterior and one posterior, instead of one cup, as in the incisor teeth. The papilla of the pulp makes

FIG. 26.

Transverse Section of a Superior Molar. [Goubaux and Barrier.]

five diverticula, which almost appear to be as many separate cavities.

In the inferior molars there are two infundibula, one occupying the middle portion of the tooth and one toward the anterior portion, which allows us to recognize a left-hand inferior molar from its right-hand homologue. The arrangement of the infundibula of the papillæ of the root is practically the same as in the superior molars.

FIG. 27.

Transverse Section of an Inferior Molar.

The molars, like the incisors, are composed of a fundamental substance and two covering layers.

*A. The Enamel* forms the bulk of the tooth. It covers the four faces and is reflected into the infundibula. On the table of a molar which has been worn down it forms the bright lines which form the Old English 𝔅.

*B. The Ivory or Dentine* is deposited on the internal face of the enamel, and finally fills up the diverticula of the pulp cavity. At first it is entirely protected by the enamel, but as the table is formed it becomes exposed, and, as it is softer than the other substance, becomes worn more rapidly and renders the wearing surface uneven and better adapted to grinding.

*C. The Cement* is very abundant on the molars. It covers the enamel, fills the infundibula of the enamel, and, in very old mouths, is often formed in excess and furnishes a new wearing surface to replace the teeth themselves, which have been destroyed by use.

ERUPTION OF THE TEETH.

*A. Eruption of the Incisors.*—The eruption of the deciduous incisors does not cause any marked constitutional phenomena, although it may cause some slight loss of appetite due, however, rather to the local pain in chewing than to any general disturbance. With the appearance of the permanent teeth the head increases in size and becomes more full, which is due principally to the space demanded for the large molars of second dentition.

The appearance of the permanent teeth is a period of general irritation of the animal economy. The first attack of periodic ophthalmia frequently appears at this time; colts seem more susceptible to contract strangles and other of the contagious diseases.

Sometimes the temporary incisors drop out before the appearance of the permanent teeth; in this case they leave a small ulcer in the gum which is followed by a swollen, red, sore point through which finally the replacing tooth appears by its internal corner, which is followed by the anterior border set, not in its future proper position, but obliquely to the axis of the jaw. When the temporary teeth have been pulled to hasten the age, as is a growing custom in some of our neighboring markets, the obliquity remains, and when found in an apparent five or six years old mouth should always be regarded with suspicion. At other times the milk teeth remain and the permanent ones protrude on their posterior border. Convulsions may occur by excessive pressure and irritation in these cases. The incisors of the upper jaw usually appear before those of the maxilla. The pincers, intermediate and corner teeth appear in succession by pairs.

*Causes which can Hasten or Retard the Eruption of the Incisors of Second Dentition.*—All ordinary horses are supposed to be born on the first of May, while thoroughbreds are supposed to be born on January 1st, and each dates its age accordingly. We may therefore have horses which officially have the same age vary from each other in their real age by some months.

Races which develop slowly, a debilitated temperament, impoverished nourishment, all tend to retard the eruption of the teeth, while on the contrary, precocious races, strong feeding, etc., hasten the appearance of the full mouth. Gestation in the young mare retards the eruption of her teeth, especially of the corner teeth, which mark the five years. In cold, damp climates the teeth are several months later in appearing than they are in warmer and dryer climates. The thoroughbred and all improved horses develop their full mouths earlier than their less well-bred relations. Exceptions may occur in both extremes. A rising three years old has been known to have all of its permanent incisors, and more frequently a six or seven years old is seen with its corner milk teeth.

*B. Eruption of the Tusks.*—The appearance of the tusks is so variable that it is of little value as an indication of age. They are usually absent in the mare, and in the horse or gelding may be out at three years, or may not pierce the gums until the animal has had all of its other permanent teeth. The *lower* tusks frequently appear almost a year in advance of the upper ones. They usually appear at about the same time as the corner incisors.

*C. Eruption of the Molars.*—As the young horse becomes older the deciduous molars become worn down until they only exist as thin plates which cap the crowns of the first three molars of permanent dentition, and are but loosely held in their alveola. They become broken, and the sharp points wound the neighboring cheek, causing difficulty of deglutition.

The two first molars of first dentition are through the gums at birth, or within a few days of it. The third appears at the end of the first month. The authorities are not in accord as to the appearance of the permanent molars, as will be seen in the following table :

| Designation of Tooth. Permanent Molars. | Epoch of Eruption. | | Designation of Tooth. | | Epoch of Eruption. Secellier. |
|---|---|---|---|---|---|
| | Girard. | Mayhew. | Inferior Molars. | Superior Molars. | |
| 4th | 10 months | 12 months | 4th | 4th | 10 and 12 mos. |
| 5th | 20 months | 18 and 24 months | 5th | 5th | 20 and 24 " |
| 1st | 30 and 32 months | | 1st and 2d | 1st | 30 and 36 " |
| 1st and 2d | | 36 months | 6th | 6th | 32 and 36 " |
| 2d and 3d | 36 months | | 3d | 2d | 40 and 42 " |
| 6th | 4 and 6 years | | | 3d | 44 and 48 " |
| 6th and 3d | | 60 months | | | |

According to Secellier the inferior milk molars fall constantly before those of the upper jaw, while the eruption of the permanent molars of both jaws takes place at the same time.

[TO BE CONTINUED.]

## Figures and Tables

**Fig. 20. f1:**
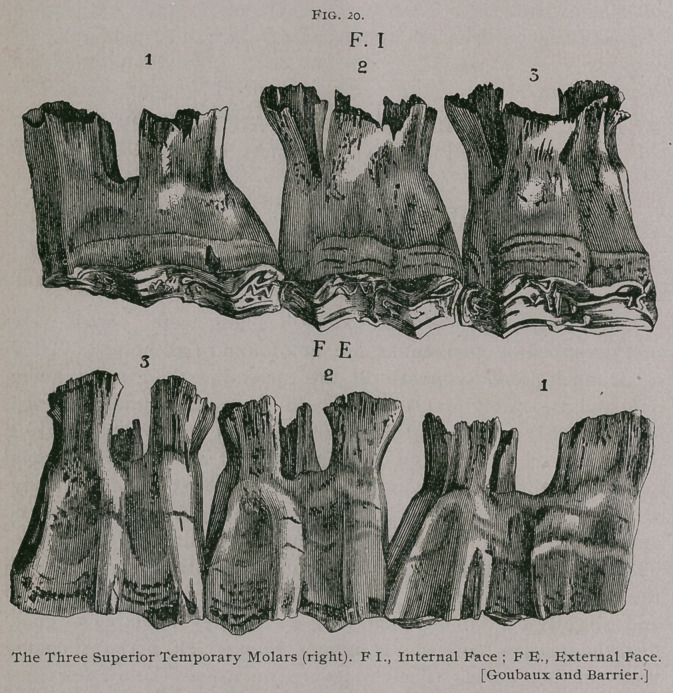


**Fig. 21. f2:**
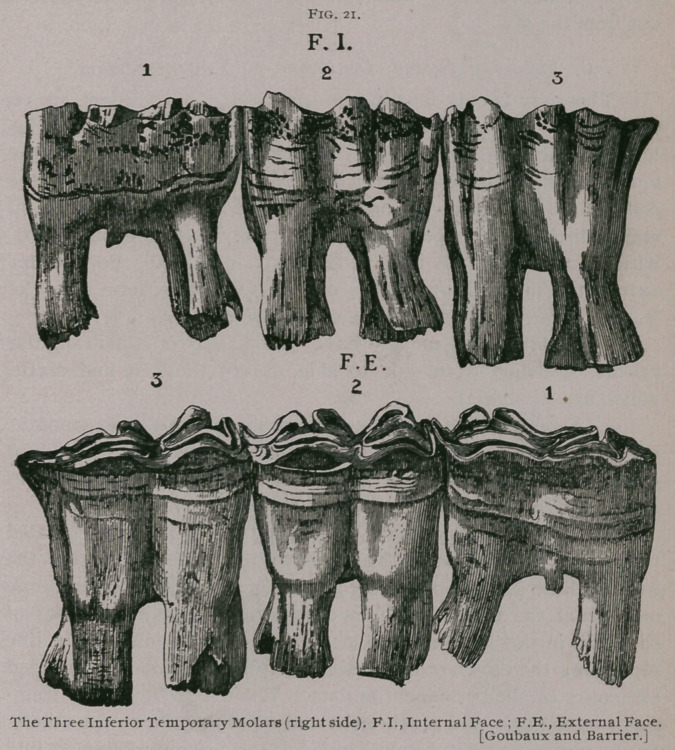


**Fig. 22. f3:**
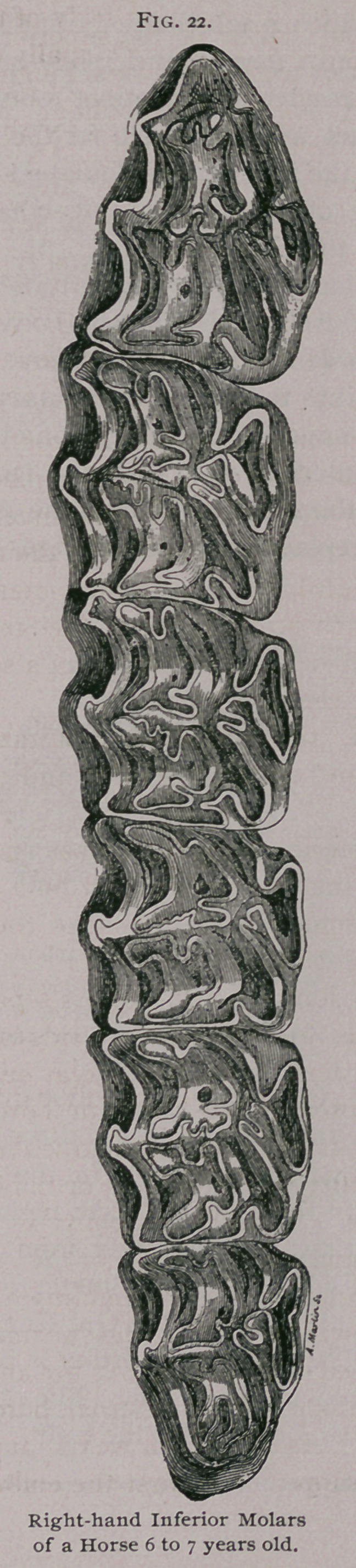


**Fig. 23. f4:**
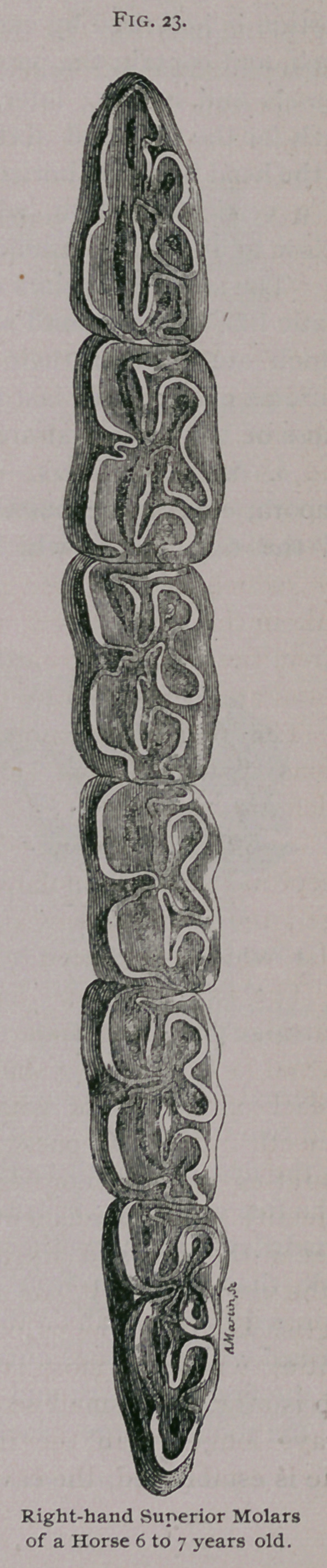


**Fig. 24. f5:**
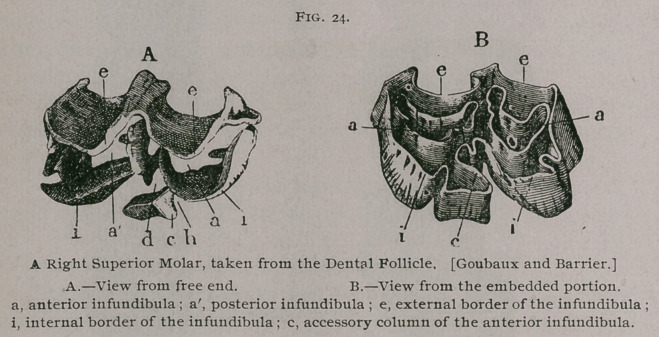


**Fig. 25. f6:**
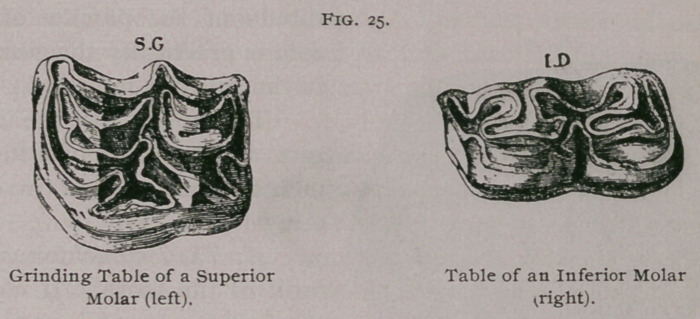


**Fig. 26. f7:**
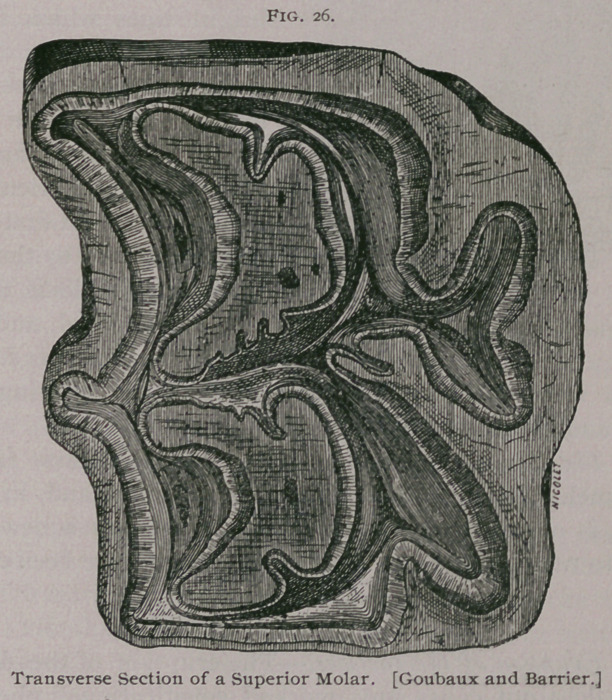


**Fig. 27. f8:**